# One year VARC-2-defined clinical outcomes after transcatheter aortic valve implantation with the SAPIEN 3

**DOI:** 10.1007/s00392-019-01461-7

**Published:** 2019-05-02

**Authors:** Costanza Pellegrini, Tobias Rheude, Teresa Trenkwalder, N. Patrick Mayr, Michael Joner, Adnan Kastrati, Heribert Schunkert, Oliver Husser, Christian Hengstenberg

**Affiliations:** 1 Klinik für Herz- und Kreislauferkrankungen, Deutsches Herzzentrum München, Technical University Munich, Lazarettstrasse 36, 80636 Munich, Germany; 2 Institut für Anästhesiologie, Deutsches Herzzentrum München, Technical University Munich, Munich, Germany; 3 Deutsches Zentrum für Herz- und Kreislauf-Forschung (DZHK) e.V. (German Center for Cardiovascular Research), Partner Site Munich Heart Alliance, Munich, Germany; 4 Department of Internal Medicine I, Cardiology, St. Johannes-Hospital, Dortmund, Germany; 5 Division of Cardiology, Department of Internal Medicine II, Medical University of Vienna, Vienna, Austria

**Keywords:** Aortic valve stenosis, Transfemoral transcatheter aortic valve implantation, VARC-2, Outcome, SAPIEN 3

## Abstract

**Aims:**

To evaluate 1-year outcome after transcatheter aortic valve implantation (TAVI) using the SAPIEN 3 (S3) prosthesis with emphasis on the composite endpoints “clinical efficacy after 30 days” and “time-related valve safety” proposed by the updated Valve Academic Research Consortium (VARC-2).

**Methods and results:**

Four hundred and two consecutive patients undergoing transfemoral TAVI with the S3 were enrolled. Mean age was 81 ± 6 years, 43% were female and median logistic EuroSCORE I was 12% [8–19]. Device success was achieved in 93% (374/402) with moderate or severe paravalvular leakage (PVL) in 2%. At 1 year all-cause mortality was 8.9% [95% CI 6.4–12.2] and new permanent pacemaker implantation rate was 16% [95% CI 12.7–20.4]. The composite endpoint time-related valve safety occurred in 29% with structural valve deterioration, defined as elevated gradients or more than moderate PVL, occurring in 13%. The clinical efficacy endpoint after 30 days was observed in 37% of patients with the main contributor symptom worsening with New York Heart Association functional class III + in 17% of cases.

**Conclusions:**

For the first time, VARC-2-defined composite endpoints at 1 year are reported and reveal a considerable proportion of patients experiencing the endpoint of time-related valve safety (29%) and clinical efficacy after 30 days (37%).

**Electronic supplementary material:**

The online version of this article (10.1007/s00392-019-01461-7) contains supplementary material, which is available to authorized users.

## Introduction

Transcatheter aortic valve implantation (TAVI) has revolutionized the treatment of symptomatic severe aortic stenosis in patients at intermediate or high risk for conventional surgical aortic valve replacement [1, 2]. With increasing operator experience, improved patient selection but also continuous evolution of transcatheter heart valves (THV) and refinement of delivery systems a considerable improvement in outcome has been achieved with a reduction in 1-year mortality from 24% with older generation THV [3] to 12% with newer generations [2].

In the case of the latest generation balloon-expandable THV, the SAPIEN 3 (S3, Edwards Lifescience, Irvine, Ca) initial results from single centres have been promising [4, 5]. Early clinical results of the Placement of Aortic Transcatheter Valves (PARTNER) II SAPIEN 3 trial have shown low 30-day mortality and low rates of stroke or paravalvular leakage (PVL) with the S3-THV [6].

Recently, longer follow-up of the PARTNER trial and the SOURCE 3 registry have become available and have confirmed excellent clinical outcome up to 1 year [7, 15]. However, the available 1-year data on this THV is limited by the fact that no study has evaluated outcomes according to the updated definitions proposed by the valvular academic research consortium (VARC-2) [9]. In these, important long-term composite endpoints regarding clinical efficacy and valve safety have been proposed. Therefore, we report 1-year outcome of a large cohort of patients treated with the S3-THV at a single centre using VARC-2 criteria and for the first time report the composite endpoints at 1 year.

## Methods

### Patient population

All patients undergoing transfemoral TAVI for severe native aortic valve stenosis with the S3-THV between January 2014 until November 2015 at the Department of Cardiology, Deutsches Herzzentrum München, Munich, Germany were included in the present analysis (*n* = 402). A multidisciplinary heart team assessed all cases taking into account the calculated perioperative risk scores as well as the patients’ characteristics at the bedside and consensus regarding the therapeutic strategy was achieved. Written informed consent was obtained prior to procedure for all patients. The 30-day outcome of a subset of the present population has been published previously [4] and for the present analysis follow-up was extended and more patients were included.

### Echocardiography and multislice computed tomography (MSCT) data analysis

MSCT was performed as part of the standard pre-procedural screening protocol. Aortic annulus measurements were assessed in multiple plane reconstructions as previously described [10]. Transthoracic echocardiography was performed before TAVI, before discharge and during follow-up at 30 days and 1 year. Data at discharge, 30 days and 1 year were available for 98.3%, 91.5% and 91.8% of surviving patients, respectively.

### Prosthesis size selection and procedure

The technical features of the S3-THV have been described elsewhere [11]. At the time of the study, the S3-THV was available in 23, 26, and 29 mm sizes. The final decision on implanted prosthesis size was left at the discretion of the physicians performing the procedure based on MSCT measurements, calcification and annulus eccentricity. Post-dilatation was performed in case of PVL II + or in case of prosthesis underexpansion.

### Definition of endpoints and follow-up

All data up to 1 year were prospectively collected during routine ambulatory visits at our outpatients’ clinic, by referring to the treating physician or other hospital documentation. Clinical endpoints were categorized using VARC-2 criteria [9]. In brief, device success was defined as absence of procedural mortality and correct positioning of a single prosthetic heart valve into the proper anatomical location and intended performance. The composite endpoint early safety at 30 days [all-cause mortality, stroke (disabling and non-disabling), life-threatening bleeding, acute kidney injury (RIFLE Stage 2 or 3 or renal replacement therapy), coronary artery obstruction requiring intervention, major vascular complication, valve-related dysfunction requiring repeat procedure] was evaluated. “Time-related valve safety” is composed of structural valve deterioration, prosthetic valve endocarditis or thrombosis, stroke and bleeding. “Clinical efficacy after 30 days” consists of all-cause mortality, disabling or non-disabling stroke, or hospitalizations for valve-related symptoms or worsening congestive heart failure (CHF). Additionally, two composite endpoints, death or readmission for heart failure and death or stroke were analyzed. Follow-up at 1 year was complete for 97.5% (392/402) and patients were censored at last event free contact.

### Statistical analysis

Continuous variables are expressed as mean with the standard deviation or the median with the interquartile range. The VARC-2 composite endpoint was assessed as time-to-event rates as were each single contributor of the composite endpoint. Additionally, to allow for assessment of possible temporal changes in categories of New York Heart Association (NYHA) functional class, transvalvular gradients and PVL during follow-up, river plots were employed. Event rates were calculated as Kaplan–Meier estimates with the respective 95% confidence intervals. A two-sided *p* value of < 0.05 was considered statistically significant for all analyses. R (version 3.3.2) was used for all analyses.

## Results

### Patient population and in-hospital outcome

The baseline characteristics of the study population are displayed in Table 1. Mean age was 81 ± 6 years, 43% were female and median logistic EuroSCORE was 12% [8–19]. Table 2 shows procedural characteristics and in-hospital outcome. The procedure was performed using conscious sedation in 51% of the cases. The 23 mm, 26 mm and 29 mm device was used in 41%, 38% and 21% of the cases, respectively. Pre-dilation was performed in the majority of cases (98%) while post-dilatation was required in 38% of procedures. Device success was achieved in 93% with PVL II + occurring in 2% (Table 2 depicts individual contributors of device success). In-hospital mortality was 0.5%.

**Table 1:** Baseline characteristics

	Total patients (*n* = 402)
Age (years)	81 ± 6
Female gender	173 (43)
Logistic EuroSCORE I	12 [8–19]
EuroSCORE II	4 [3–7]
Society of thoracic surgeons score	4.3 [2.7–6.6]
New York Heart Association class III/IV	248 (62)
Chronic obstructive pulmonary disease	58 (14)
Diabetes mellitus	124 (31)
Glomerular filtration rate (ml/min)	54 ± 22
Peripheral vascular disease	53 (13)
Previous stroke major/minor	39 (10)
Previous pacemaker	41 (10)
Previous myocardial infarction	41 (10)
Previous percutaneous coronary intervention	170 (42)
Previous coronary artery bypass graft	24 (6)
Echocardiographic characteristics	
Left ventricular ejection fraction < 35%	40 (10)
Mean transaortic gradient (mmHg)	44 ± 16
Pulmonary arterial pressure ≥ 60 mmHg	34 (9)

All data are mean ± standard deviation, median [interquartile range] or absolute number (percentage)

**Table 2:** Procedural characteristics and in-hospital complications

	Total patients (*n* = 402)
Procedural characteristics	
Conscious sedation	203 (51)
Pre-dilatation	392 (98)
Post-dilatation	154 (38)
Procedural time (min)	58 ± 29
Contrast (ml)	118 ± 58
Fluoroscopy time (min)	13 ± 6
Device success^a^	374 (93)
Procedural mortality	2 (0.5)
Correct position	400 (99.5)
Intended performance^b^	378 (94)
PVL II +	8 (2)
Elevated gradient (≥ 20 mmHg)	13 (3)
Multiple valves	4 (1)
Conversion	3 (0.7)
In-hospital characteristics	
Days on Intensive Care Unit	1 [1–2]
Days in hospital	5 [4–6]
ln-hospital mortality	2 (0.5)
All stroke	8 (2)
Major vascular complication	31 (8)
Life-threatening bleeding	20 (5)
Renal failure (AKIN 2/3, including dialysis)	12 (3)
Coronary artery obstruction w/PCI	1 (0.2)
Myocardial infarction	1 (0.2)

^a^Multiple events possible

^b^No patient-prosthesis mismatch, mean aortic valve gradient < 20 mmHg or peak velocity < 3 m/s, without moderate or severe prosthetic valve regurgitation of the first implanted prosthesis

### Clinical outcomes during 1 year after TAVI

**Fig. 1:**
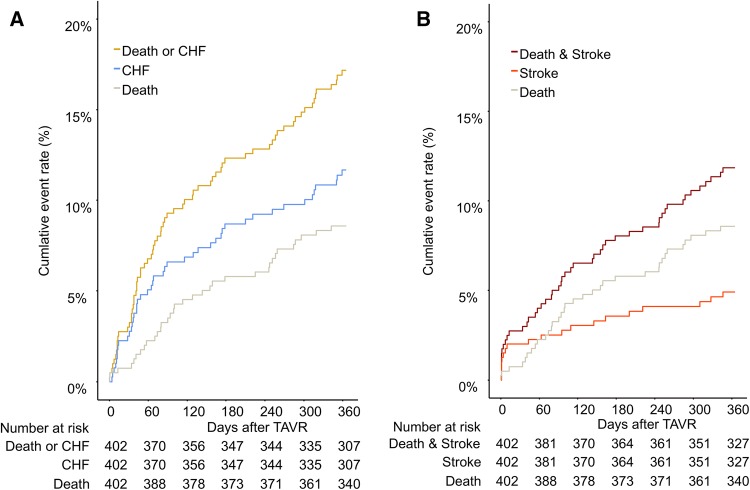
Cumulative incidence of death and CHF (**a**) and death and stroke (**b**). Kaplan–Meier failure curves for the cumulative event rate of death and/or CHF (**a**) and death and/or stroke (**b**) during the first year after TAVI

All-cause mortality at 30 days was 0.8% and increased to 8.9% at 1 year (Table 3; Fig. 1). At 30 days and 1 year, rate of readmission for CHF was 2.5% and 12.0%, respectively. The 1-year composite of all-cause death or readmission for CHF was 18% (Fig. 1a). Cumulative stroke rate at 1 year was 5% with 2% occurring within the first 30 days. The 1-year composite of all-cause death or stroke was 12% (Fig. 1b). The cumulative incidence of permanent pacemaker implantations (PPI) in pacemaker-naive patients was 12.8% at 30 days and increased to 16.2% at 1 year.

**Table 3:** Cumulative Kaplan–Meier event rates at 30 days and at 1 year

	30 days		1 year	
	KM estimate (%) [95% CI]	Events (*n*)	KM estimate (%) [95% CI]	Events (*n*)
All-cause mortality	0.8 [0.2–2.3]	3	8.9 [6.4–12.2]	34
Cardiac mortality	0.75 [0.24–2.31]	3	5.2 [3.66–8.40]	21
All stroke	2.0 [1.0–4.0]	8	5.0 [3.2–7.8]	19
Major vascular complication	7.8 [5.5–10.9]	31	8.3 [6.0-11.5]	33
Life-threatening bleeding	6.0 [4.1–8.8]	24	9.0 [6.6–12.3]	35
Renal failure (AKIN 2/3, including dialysis)	3.0 [1.7–5.2]	12	3.6 [2.1–5.9]	14
Percutaneous coronary intervention	0.3 [0.04–1.8]	1	1.9 [0.9–3.9]	7
Myocardial infarction	0.3 [0.04–1.8]	1	1.4 [0.6–3.2]	5
New permanent pacemaker implantation^a^	12.8 [9.8–16.7]	46	16.2 [12.7–20.4]	57
Valve-related dysfunction w/ BAV, TAVR or SAVR	0	0	0.3 [0.04–1.9]	1
Valve-related dysfunction^b^	5.4 [3.5–8.1]	21	20.6 [15.3–27.5]	52
Endocarditis	0	0	1.9 [0.9-4.0]	7
Congestive heart failure w/ hospitalization	2.5 [1.4–4.6]	10	12.0 [9.1–15.7]	45
Early safety (at 30 days)^b^	13.7 [10.7–17.4]	55	–	–
Clinical efficacy (after 30 days)^b^	–	–	37.2 [32.2–42.7]	133
Time-related valve safety^b^	12.8 [9.9–16.5]	51	29.4 [24.7–34.7]	105

*AKIN* acute kidney injury, *BAV* balloon aortic valvuloplasty, *SAVR* surgical aortic valve replacement, *TAVR* transcatheter aortic valve replacement

^a^Only pacemaker-naive patients

^b^For definition of composite endpoints, see Methods

### Temporal course of NYHA class

**Fig. 2:**
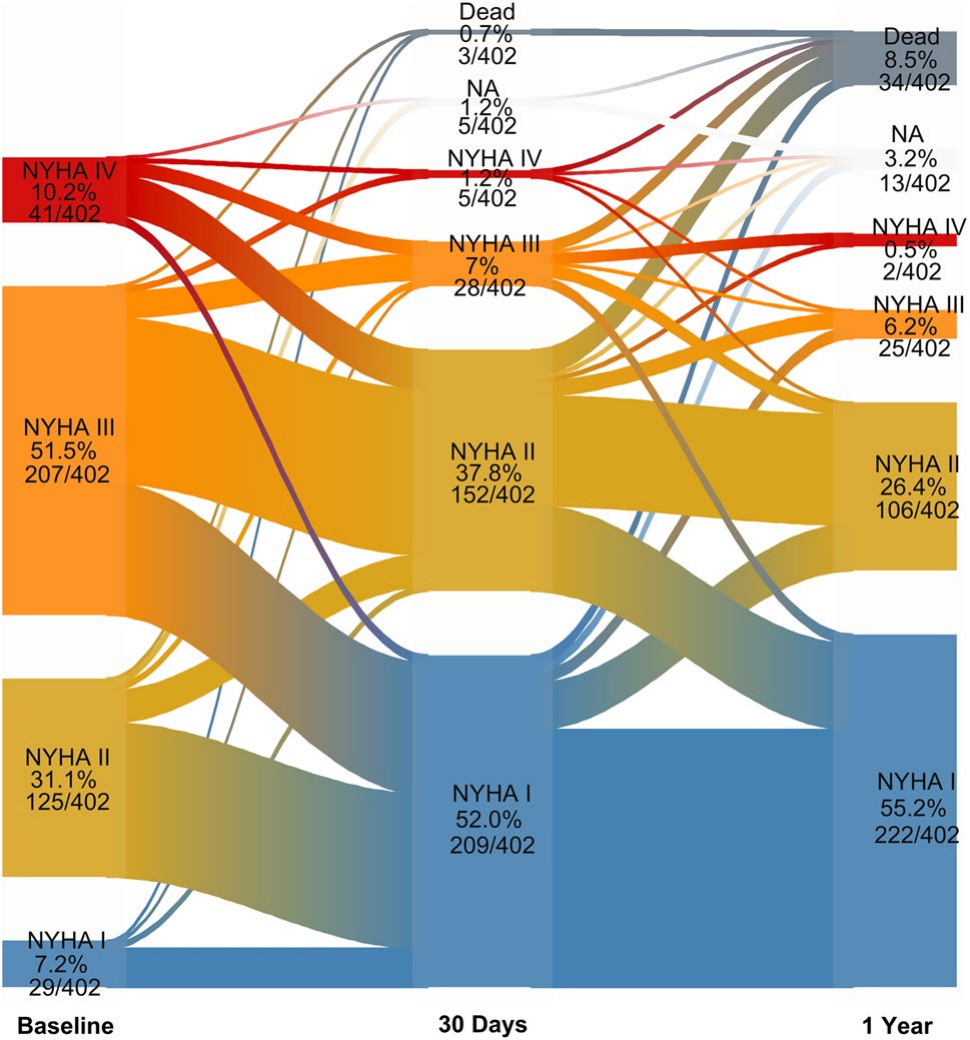
New York Heart Association Functional Class at baseline and during follow-up. Change in NYHA categories during the first year after TAVI

Figure 2 shows the river plot of changes in NYHA categories. Overall, 52% and 55% of the patients were asymptomatic (NYHA I) at 30 days and 1 year, respectively. Within 1 year, 73% of patients improved at least in one functional class, 13% experienced no change and only 3% worsened. In 11.7% of cases, NYHA class at 1 year was not available due to death (8.5%) or was missing (3.2%).

### Echocardiographic follow-up

**Fig. 3:**
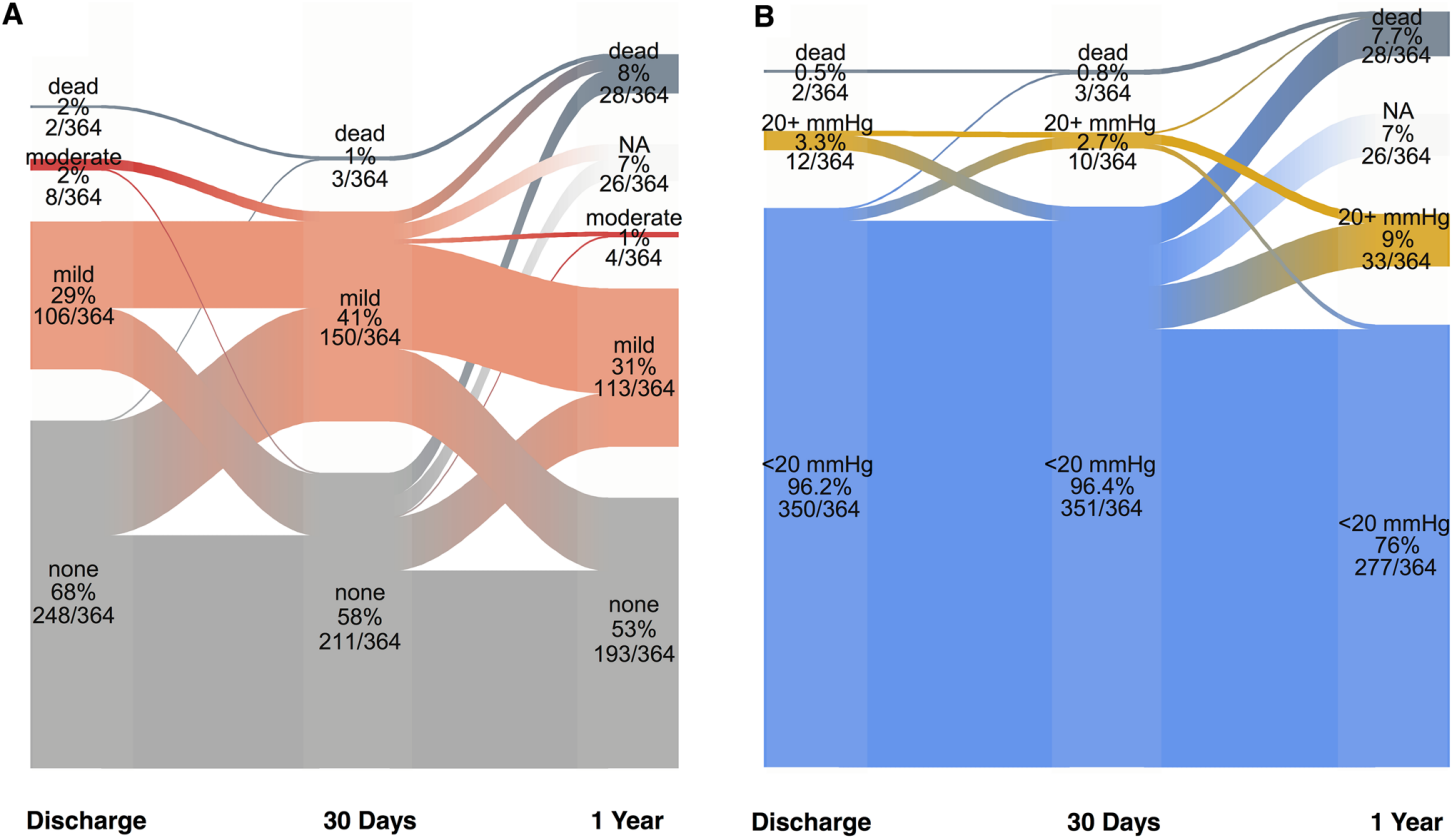
Echocardiographic valve performance after discharge for PVL (**a**) and transvalvular gradients (**b**). Change in PVL (**a**) and elevated gradients during the first year after TAVI. Note that only patients with complete echocardiography at discharge and 30 days or dead (*n* = 364) are displayed

Mean transaortic gradients before TAVI, at discharge and during follow-up are displayed in Online Resource 1, showing stable mean gradients around 12 mmHg. The proportion of patients with elevated gradients (≥ 20 mmHg) and moderate PVL and its course during follow-up is depicted in Fig. 3. Of patients with complete echocardiography at discharge and 30 days or with known mortality status (*n* = 364), PVL was moderate in 2% at discharge and 1% at 1 year. There was no patient with severe PVL (Fig. 3a). The proportion of patients with elevated gradients was 3.3%, 2.7% and 9% at discharge, 30 days and 1 year, respectively. Patients with elevated gradients at discharge had significantly smaller aortic annuli compared to those without elevated gradients (3.7 ± 0.5 vs. 4.8 ± 0.9 cm^2^; *p* < 0.001), were more often female (84.6% vs. 41.6%; *p* = 0.002) and were all treated with the 23 mm prosthesis. Figure 3b shows a considerable increase in elevated gradients from 30 days to 1 year.

### VARC-2-defined composite endpoints

**Fig. 4:**
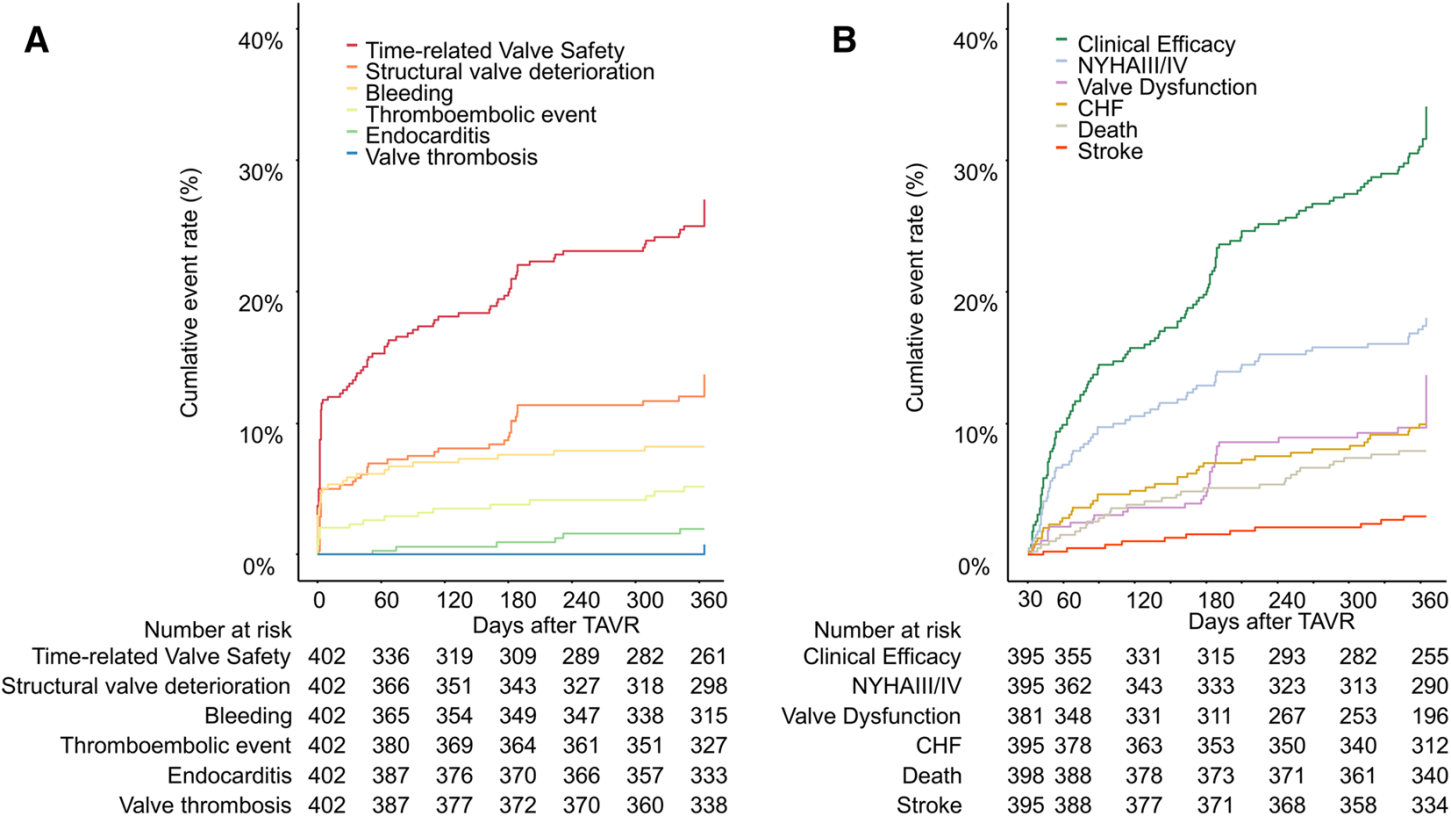
VARC-2 composite endpoints: time-related valve safety (**a**) and clinical efficacy after 30 days (**b**). Kaplan–Meier failure curves for the cumulative event rates of time-related valve safety (**a**) and clinical efficacy after 30 days (**b**) with rates of their respective contributors

The combined early safety endpoint at 30 days occurred in 13.7%. During the first year after TAVI, 29.4% experienced the time-related valve safety endpoint (Table 3). Figure 4a shows the individual contributors to this endpoint, the main contributor being structural valve deterioration, defined as elevated gradients (≥ 20 mmHg) or PVL II + with a cumulative incidence of 12.9% at 1 year. The clinical efficacy endpoint after 30 days was observed in 37.2% (Fig. 4b). The main contributor of this composite endpoint was symptom worsening (NYHA III/IV) with a cumulative incidence of 17.2%.

## Discussion

In a contemporary population of TAVI patients who were treated in a single centre with the S3-THV, we found excellent results for 1-year mortality. For the first time, we report VARC-2-defined composite endpoints at 1 year, namely “clinical efficacy after 30 days” and “time-related valve safety”.

### The VARC endpoint definitions—1-year on the SAPIEN 3 transcatheter heart valve

The updated Valve Academic Research Consortium criteria provide a standardized framework for evaluation and comparison of clinical outcomes after TAVI [9]. Although the adoption of VARC criteria has been increasing over time, a considerable number of publications does not report outcomes according to VARC [12, 13]. Indeed, even in recent pivotal TAVI trials, while applying VARC-2 criteria for reporting in-hospital outcomes, important composite endpoints such as device success and early safety at 30 days are not reported [6–8].

The S3-THV is widely used, however, relatively little data on 1-year results is available and no data at all is available on the VARC-2 composite endpoints. The majority of data come from the PARTNER II trial [6], in which 952 patients treated transfemorally from the intermediate-risk population presented an all-cause mortality of 12.3% and the combined rate of all-cause mortality and stroke was 17.2% at 1 year [14]. Recently, 1-year data from the SOURCE 3 and the Israeli TAVR registry showed even lower all-cause mortality rates at 1 year ranging from 8.5 to 12.6% and a stroke rate of 3.1% [15, 16]. A recent sub-group analysis of the SOURCE 3 registry showed mortality rates of 9.3% in patients aged 75–80 years [17], while Eichler et al. [18] presented all-cause mortality rates of 13.8% at 1 year. Our results from 402 patients are comparable favourably to this recent 1-year data with an all-cause mortality of 8.9% and stroke rate of 5.0%.

Very recently, results from randomized trials of a low-risk TAVI population have been published in the New England Journal of Medicine, showing even lower 1-year mortality rates ranging from 1–2.4% [19, 20]. These promising results further strengthen the positive results of TAVI and encourage to a further expansion to a younger and low-risk population.

### VARC-2 composite endpoints

The composite endpoint “device success” is an important measure of acute procedural success and few studies have assessed this using the S3-THV. Our group has previously published 30-day outcomes using the S3-THV [4]. In this extended analysis with 1-year follow-up and a significant increase in sample size, we were able to show stable rates of device success (93% vs. 97.6%) and early safety at 30 days (13.7% vs. 10%). As far as clinical efficacy after 30 days and time-related valve safety is concerned, little data are available in the current literature and with other THV [21]. In the present study, we found a relatively high incidence of these endpoints mostly driven by symptomatic heart failure (NYHA class III/IV) or valve-related dysfunction with elevated gradients.

Frequently, clinical conclusions are drawn from comparison of summary data. From those, it is almost impossible to follow the development of certain parameters. Here, we created river plots for NYHA class and echocardiographic parameters to better understand the effects of TAVI on both, the individual and the population. Using a river plot-based analysis, we observed a dynamic change in elevated gradients calling into question the clinical significance of this finding. Although the mean of mean pressure gradients was low throughout the first year (12 mmHg), a considerable proportion of patients (9%) exhibited elevated gradients at 1 year. Other groups have reported even higher rates of patient-prosthesis mismatch (24%) mostly due to elevated gradients with the S3-THV [22]. In this analysis, patients experiencing elevated gradients displayed no significant difference in outcome in terms of mortality, stroke rates or worsening of symptoms. A previous study on surgical aortic valve replacement suggested higher rates of re-intervention in patients with elevated gradients, especially in younger patients [23]. Moving towards a younger TAVI population, assessing the impact of elevated gradients on valve durability is of the utmost importance and future studies in large populations with extended follow-up are warranted to fully assess the significance of this finding.

Recently, it has become evident that not only valve deterioration but also valve thrombosis does contribute to elevated gradients [24]. Subclinical leaflet thrombosis, a phenomenon relatively recently recognized in the field of TAVI [25], may be a possible explanation for the considerable dynamic in the rate of elevated gradients. In the current analysis, we detected only three cases of valve thrombosis; however, this population treated from 2014 to 2015 was not routinely screened for valve thrombosis with serial examinations by CT or transesophageal echocardiography. Hence, the incidence of valve thrombosis may be underestimated and cannot be excluded as temporary or longer lasting cause of elevated gradients.

### New permanent pacemaker implantations

Cardiac conduction disturbances leading to PPI are a frequent and important complication after TAVI. Although earlier investigations found no negative effect of new PPI on outcome [26], recent data have identified chronic pacing as independent predictor of 1-year mortality after TAVI and as an important cause of prolonged hospital stay [27].

In the case of the S3-THV, first systematic data on PPI showed incidences of 13% until up to 25.5% [28–30]. This led to several investigations examining in more detail the potential underlying mechanisms and demonstrated PPI rates of 11.6% [14], 13.1% [6], and 16% [10] at 30 days in pacemaker-naive patients. Multiple factors have been described to predict PPI following TAVI, especially a previous right bundle branch block [31–33]. In an extended meta-analysis of PPI following TAVI, Siontis et al. [34] categorized these factors into patient-related, electrocardiographic and procedural factors. While the former two categories cannot be influenced by the operator’s choices or skills, device-related factors may be influenced by sizing strategies, implantation technique and implantation depth. Development of novel devices should particularly address these “modifiable” features to allow for less need of PPI after TAVI.

### Limitations

This is an observational study from a single centre without centre-independent adjudication of postprocedural results and lack of independent echocardiographic core lab assessment. Clinical benefit was assessed by NYHA functional class and may be patients’ subjective perception.

## Conclusions

The present study assesses 1-year outcomes with the S3-THV according to VARC-2-defined endpoints with low rates of mortality and stroke at 1 year. For the first time, VARC-2-defined composite endpoints at 1 year are reported and reveal a considerable proportion of patients experiencing the composite endpoint of time-related valve safety (29%) and clinical efficacy after 30 days (37%). The main contributor to these combined endpoints was elevated gradients. Further research is warranted to reveal the underlying mechanisms behind this observation.

## Electronic supplementary material

Below is the link to the electronic supplementary material.

Mean transaortic gradients before and after TAVI. Mean transaortic gradients before TAVI and during follow-up. (DOCX 25 KB)
