# Supplementary figures and images for: One year VARC-2-defined clinical outcomes after transcatheter aortic valve implantation with the SAPIEN 3
Source: Clin Res Cardiol. 2019 May 2;108(11):1258–65. doi: 10.1007/s00392-019-01461-7 (PMC6805964; doi:10.1007/s00392-019-01461-7)

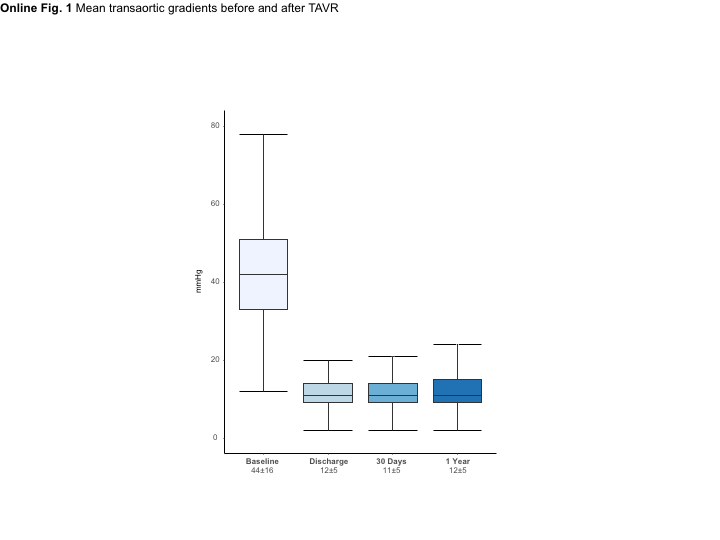

Supplement: Supplementary file 1 — Mean transaortic gradients before and after TAVI. Mean transaortic gradients before TAVI and during follow-up. (DOCX 25 KB) [file 392_2019_1461_MOESM1_ESM.docx]
